# From microsatellites to single nucleotide polymorphisms for the genetic monitoring of a critically endangered sturgeon

**DOI:** 10.1002/ece3.5268

**Published:** 2019-06-11

**Authors:** Séverine Roques, Emilie Chancerel, Christophe Boury, Maud Pierre, Marie‐Laure Acolas

**Affiliations:** ^1^ Aquatic Ecosystems and Global Changes IRSTEA, EABX UR Cestas France; ^2^ UMR 1202 BIOGECO INRA Cestas France

**Keywords:** *Acipenser sturio*, conservation program, high‐throughput sequencing, inbreeding, parentage assignment, relatedness

## Abstract

The use of genetic information is crucial in conservation programs for the establishment of breeding plans and for the evaluation of restocking success. Short tandem repeats (STRs) have been the most widely used molecular markers in such programs, but next‐generation sequencing approaches have prompted the transition to genome‐wide markers such as single nucleotide polymorphisms (SNPs). Until now, most sturgeon species have been monitored using STRs. The low diversity found in the critically endangered European sturgeon (*Acipenser sturio*), however, makes its future genetic monitoring challenging, and the current resolution needs to be increased. Here, we describe the discovery of a highly informative set of 79 SNPs using double‐digest restriction‐associated DNA (ddRAD) sequencing and its validation by genotyping using the MassARRAY system. Comparing with STRs, the SNP panel proved to be highly efficient and reproducible, allowing for more accurate parentage and kinship assignments' on 192 juveniles of known pedigree and 40 wild‐born adults. We explore the effectiveness of both markers to estimated relatedness and inbreeding, using simulated and empirical datasets. Interestingly, we found significant correlations between STRs and SNPs at individual heterozygosity and inbreeding that give support to a reasonable representation of whole genome diversity for both markers. These results are useful for the conservation program of *A. sturio* in building a comprehensive studbook, which will optimize conservation strategies. This approach also proves suitable for other case studies in which highly discriminatory genetic markers are needed to assess parentage and kinship.

## 1 INTRODUCTION

Many threatened species are managed under captive breeding programs that prioritize the retention of sufficient and representative genetic variation of the original population and the avoidance of inbreeding in the future generations (Ballou & Lacy, 1995; Fraser, 2008; Jamieson & Lacy, 2012). This is generally obtained through genetic monitoring, usually parentage testing and accurate estimations of relatedness and inbreeding (Russello & Amato, 2004). These programs need precise genetic data, critical to guide breeding schemes and manage restocking efforts. For example, the evaluation of restocking success is highly dependent on reliable parentage assignment of recaptured progeny issued from the captive program (Roques, Berrebi, Chèvre, Rochard, & Acolas, 2016; Schreier, Stephenson, Rust, & Young, 2015). Also, breeding schemes usually prioritize the less related parent pairs, with this last approach being an efficient way to minimize kinship or inbreeding at each generation (Ivy & Lacy, 2012; Ivy, Putnam, Navarro, Gurr, & Ryder, 2016). So far, microsatellites or short tandem repeats (STRs) have been the most widely used molecular markers in captive breeding programs for parentage and relatedness assessment, because of their high polymorphism and multiallelic state. However, when higher resolution is needed, the addition of many more STR markers requires a substantial investment of time to ascertain reliability. Due to these limitations and thanks to recent advances in “genomics” technologies, such as next‐generation sequencing (NGS) and high‐throughput genotyping (Metzker, 2010; Tsuchihashi & Dracopoli, 2002), STRs are now being largely replaced by single nucleotide polymorphisms (SNPs). Genome‐wide markers such as SNPs offer several advantages over others markers, such as abundance in the genome and low mutation rates (Morin, Luikart, Wayne, & the SNP workshop group, 2004), and may provide most representative patterns of the entire genome (Vali, Einarsson, Waits, & Ellegren, 2008). Also, aside from the low cost in genotyping a great number of individuals and markers, they are highly reproducible, reliable, and easily transferrable between laboratories, all of these qualities being the panacea for long‐term conservation programs. Single nucleotide polymorphisms have proven to be especially powerful tools in species for which diversity is low, because their number can be increased until optimum resolution is needed (Kleinman‐Ruiz et al., 2017). Indeed, in the last ten years, an increasing number of papers have described the development of novel SNP markers and their comparison to STR markers. In most cases, the novel sets of SNPs outperform STRs to assess identity, parentage (Tokarska et al., 2009; Wright et al., 2015), or population structure (Glover et al., 2010; Senn et al., 2013). However, while studies have mostly focused on SNP efficiency for parentage assignment at different biological scales (species, populations, and individuals), there are still few papers evaluating SNP sensitivity for kinship or inbreeding estimation in different kin contexts (Kopps, Kang, Sherwin, & Palsbøll, 2015; Thrasher, Butcher, Leonardo Campagna, Webster, & Lovette, 2018). The performance of genetic markers for estimating relatedness and inbreeding is especially of high concern for the conservation of captive populations. The reliability of these parameters is usually dependent on the number and variability of markers available, as well as the kin composition and/or demographic history of the populations or species (Miller, Buchner, et al., 2014; Miller, Malenfant, et al., 2014). Because management actions are often taken based on genetic advice, there is a great interest to evaluate differences and possible ascertainment bias between STR and SNP markers.

The European sturgeon (*Acipenser sturio*) survives in Western Europe where only a single relict natural population occurs in the Gironde–Garonne–Dordogne watershed in France. This population has been supported since 1995 by a breeding and restocking program (Williot, Rouault, & Brun, 2011), by which a large number of larvae and juveniles (>1.5 million) have been released in the rivers (from 2007 to 2017). Genetic monitoring of the species started in 2009 based on a set of 18 STRs (Roques et al., 2016) thanks to a regular monitoring of the estuarine fraction of the population (Acolas, Roqueplo, Rouleau, & Rochard, 2011). Results overall showed that *A. sturio* genetic diversity is low and that the genetic heterogeneity found in the initial broodstock was maintained in the sustained population (Roques, Berrebi, Rochard, & Accolas, 2018). Parentage assignments of juveniles issued from restocking further indicated that most individuals (95%) captured in the Gironde estuary are issued from the restocking program (Roques et al., 2016) and that a small proportion of these fish (around 10%) hold some level of inbreeding. These results support the rarity of reproduction in the wild and highlight the need for a careful genetic management of captive‐born generations both in the captivity and in the wild, for the successful recovery of the species. By 2020/2022, the individuals produced from restocking (7 generation F1 ex‐situ since 2007) should be old enough to reproduce and resulting in F2 generations. There will be in the rivers F1 and F2 offspring released by the continuous restocking program (issued from the captive stock) as well as potential F1 and F2 offspring from natural reproduction (i.e., mostly issued from F1 releases). It will then be necessary to be able to identify the F1 parents of any of these F2 descendants. This information is important, because the success of the restocking program and the sustainability of the in situ population will be validated only if we can demonstrate that these released individuals reproduce successfully in the natural environment. The difficulty lies in the fact that current species diversity is low and on the need to identify more and more genetically similar individuals, as we expect a loss of diversity in these future cohorts. The capacity of resolution of the microsatellite markers might be quickly limited, thus requiring a new tool as informative and efficient as possible.

To improve the genetic management of this species, we thus envisaged the development of SNP markers through NGS. There are usually two main approaches aimed to discover novel SNPs, mostly depending on the availability of genomic resources. The easiest way is when nonmodel species are closely related to model organisms for which a large amount of genomic information and/or SNP chips are already available and could be “cross‐amplified” (e.g., Cooper, Miller, & Kapuscinski, 2009; Haynes & Latch, 2012; Ogden, Baird, Senn, & McEwing, 2012). However, for the production of genomic data for any species (i.e., with no reference and/or phylogenetically closed genome), restriction site‐associated DNA sequencing (RADseq) has proved to be a powerful tool for SNP discovery and genotyping (Baird et al., 2008; Davey, Davey, Blaxter, & Blaxter, 2010; Leitwein et al., 2016; Rowe, Renaut, & Guggisberg, 2011).

While the high cost of sequencing has long been considered the main drawback of this method, some modifications of the original RAD protocol (e.g., double‐digest RADseq and ddRADseq) have recently increased time and cost efficiency (Peterson, Weber, Kay, Fisher, & Hoekstra, 2012; Puritz et al., 2014). Once SNPs have been discovered, filtering and validation steps further aim to select a valuable set that depends on the application being needed or the hypothesis being tested. Recent studies have underlined that genotyping errors inherent to NGS approaches are one of the most important factors to take into account during validation (reviewed in Hohenlohe, Catchen, & Cresko, 2012; Mastretta Yanes et al., 2015; Ogden et al., 2013). The recent literature on the subject thus converges on the importance of quantifying biases and limitations inherent to each method (Shafer et al., 2017).

In this study, we characterized novel SNP markers for the critically endangered *A. sturio*. In this remnant population composed of individuals being genetically related, we compared the effectiveness of these novel SNP markers with microsatellite markers in respect to parentage assignment, and for relatedness and inbreeding estimations. Specific aims were to (a) detect novel SNP markers for the species using double‐digest sequencing protocol for Ion Torrent sequencing (Life technologies; Ion Proton, Ion PGM) and select an optimal set for parentage assignment through validation by genotyping using the MassARRAY system, (b) apply the novel set of markers for parentage assignment of 192 juveniles of the French captive stock, for which putative parents are recorded in the breeding database, (c) compare the resolution of SNPs and STRs for parentage testing and for their effectiveness in measuring genetic diversity, relatedness, and inbreeding, for empirical and simulated datasets, and (d) define relevant and precise genetic indicators for the long‐term monitoring of the breeding and restocking program of *A. sturio* (Studbook implementation) that can be transposed to other sturgeon species.

## 2 MATERIALS AND METHODS

### 2.1 Sampling

Fin samples were obtained for 275 *A. sturio*. Samples included 33 wild‐born breeders, 8 captive‐born adults (F1 cohort of 1995 issued from a single pair of relatives; F1‐1995), 40 juveniles captured by trawling in the Gironde estuary (Aquitaine, France) during 2009–2014 population monitoring campaigns (Acolas et al., 2011), and 195 F1 juveniles (JUV) kept in captivity. Among recaptures, 40 samples were previously analyzed by STRs (Roques et al., 2016). All juveniles from JUV have whole (both parents known) or partial (the mother is known but two or three fathers are possible) breeding records. They are issued from a total of 22 different families (2007–2011 cohorts). For all samples, DNA extraction of fins collected and preserved in 95% ethanol was carried out using the DNA Tissue and Blood extraction kit, following the manufacturer protocol (Qiagen).

### 2.2 ddRAD sequencing and SNP discovery

Forty samples were used for double‐digest RADseq‐ion library preparation: 33 wild‐born breeders and 6 F1 offspring (parents are known). Two technical replicates were included to estimate repeatability and error rates resulting from library preparation, sequencing, and bioinformatic analyses. Replicates are issued from the same DNA source but were processed independently. DNA quantification was made using the Quant‐iT dsDNA BR Assay (Thermo Fisher Scientific) according to the manufacturer's instructions. ddRADseq library preparation protocol followed the methods described by Pukk, Kisand, Ahmad, Gross, and Vasemagi (2014), with some modifications. 500 ng of DNA was digested for 2 hr at 37°C with two rare‐cutting restriction enzymes, 10 U of AseI and PstI (New England Biolabs). After magnetic bead‐based purification (CleanNA, 1.6× ratio), ligation was done with 8 µl of digested DNA, 0.5 mM of ATP, 1× of T4 DNA ligase buffer, 800 U of T4 DNA ligase (NEB), and 0.04 µM of P1‐AseI and A‐PstI adapters. To differentiate the 40 samples, 10–12 bp barcodes were added on the A‐PstI adapter to access barcodes associated with each sample (see Appendix S1). The 20 μl ligation reactions were carried out at 22°C for 2 hr, heat‐inactivated for 11 min at 65 C, and cooled at 19°C (1°C/min). Libraries were purified with beads (CleanNA, 1.8× ratio) and quantified with the Ion Library TaqMan Quantitation Kit (Thermo Fisher Scientific) before equimolar library pooling. Size selection was made on the pool (30 µl) using automated size‐selection technology, Pippin Prep (Sage Science; 2% agarose cartridge; 300 pb, “tight” mode) and purified using magnetic beads (CleanNA, 1.6× ratio). 30 µl of the sized pool was amplified in 100 µl reaction using 1× Q5 High Fidelity PCR Master mix (New England Biolabs) and 0.6 µM of primers A and P1 (New England Biolabs). PCR consisted of 98°C for 30 s followed by 10 cycles of 98 C for 10 s, 58°C for 30 s, and 65°C for 30 s. A final purification on magnetic beads (CleanNA, 1× ratio) was made on the amplified pool. Quality and quantity assessment were done using High Sensitivity DNA kit on Bioanalyzer 2,100 (Agilent Technologies). Emulsion PCR and enrichment were performed on Ion OneTouch 2 System (Thermo Fisher Scientific), according to the manufacturer's instructions. The libraries were loaded on an Ion Proton I Chip and sequenced with an Ion Proton System (Thermo Fisher Scientific) at the Genome Transcriptome Facility of Bordeaux, France.

All the raw sequences were quality‐trimmed using the default settings of the Ion Torrent BaseCaller (>Q16 with a windows size of 30 bases) and demultiplexed based on their barcodes. Then, stacks (Catchen, Hohenlohe, Bassham, Amores, & Cresko, 2013) were used to trim the reads to a length of 200 bp (“process_radtags” program) and to identify putative SNPs (“denovo_map” program) with the following parameters: minimum number of identical reads = 6, number of mismatches allowed between loci when processing a single individual = 2, and number of mismatches allowed between loci when building the catalogue = 3. For constraints imposed by the Mass Array primer design protocol, RAD tags containing more than one SNP were discarded and we removed SNPs located <20 bp and >179 bp in the 200 bp sequence.

### 2.3 Single nucleotide polymorphism quality, filtering, and genotyping

Single nucleotide polymorphism filtering consisted of (a) the selection of SNPs present in more than 50% of individuals (*N* = 20) and with a minor allele frequency (MAF) ≥ 0.175 (e.g., Roesti, Salzburger, & Berner, 2012); (b) the conservation of SNPs with the same genotype between the two replicates; (c) the elimination of SNPs showing Mendelian inconsistencies between parent and offspring genotypes from two sturgeon families (i.e., two parent pairs named VINTCENTMAI * JUSTIN and FRANCINE * MARTINIEN, with two and four descendants, respectively); (d) the conservation of SNPs showing sufficient proportions of heterozygotes in the population, and (e) elimination of RAD tags differing by just a single insertion (considering by STACKS as two different loci).

Finally, 186 candidate SNPs were submitted for assay design using the MassARRAY^®^ Assay (see Appendix S2 for details on filtering steps) Design version 3.1 (Agena Biosciences, Hamburg, Germany). Four multiplexes consisting of 154 SNPs were retained (Appendix S3). Genotyping was performed with the Agena Biosciences technology following standard protocols (Gabriel, Ziaugra, & Tabbaa, 2009). Analysis was carried out on individuals in the captive stock: *N* = 33 wild‐born breeders, *N* = 4 captive‐born adults (F1‐1995), *N* = F1 captive‐born juveniles, and *N* = 40 recaptured individuals in the Gironde estuary. We included two exogenous positive controls and two negative (water) controls to check reliability. Raw data analyses were performed using the software MassARRAY TYPER 4.0. We filtered out monomorphic SNPs (i.e., declared as polymorphic SNPs from ddRADseq but proved to be monomorphic after validation by MassArray), loci with weak or ambiguous signal (loci displaying more than three clusters or unclear cluster delineation), and those with Mendelian discordance (checked in 11 captive‐born families).

### 2.4 Individual identification and parentage assignment

We then tested our SNP panel for individual identification and parentage assignment and compared its efficiency to STRs. For individual identification, the probability of identity (PID, the probability that two individuals hold the same genotype) was assessed and compared to that of STRs, based on the same *N* = 37 breeders. To test for the resolution of the panel of SNPs in our context, calculations were also done based on the SNP allelic frequencies of the future breeders (PID Juv; *N* = 148 related and nonrelated individuals, captive‐born in 2007 and 2008 that will be mature in the next years). The combined PID over loci was calculated using the analysis module in GenAlEx 6.5 for unrelated individuals (PID‐unrel) or relatives (i.e., siblings and PID‐sibs) (Peakall & Smouse, 2012).

For parentage assignment, the program CERVUS (Kalinowski, Taper, & Marshall, 2007) uses a likelihood‐based approach to assign parental origin combined with simulation of parentage analysis to determine the confidence of parentage assignments. Simulations were run in CERVUS to determine the distribution of the critical values of Delta or LOD score for 80% and 95% confidence levels. Simulation parameters were set as detailed in Roques et al. (2016). In total, true paternity was screened on 40 captured and 192 captive juveniles. Both parents were known for 128 F1 juveniles (i.e., to test reliability) whereas for the remaining individuals (*N* = 67), female identity is recorded but two or three males were possible (this is because reproduction is assisted and several male gametes were mixed in some cases). In this case, male ID deduced by parentage testing will be recorded in the captive breeding database. Only those parents showing 95% Trio confidence and 0/1 mismatch with their putative offspring were validated as “true” parents. To compare parentage efficiency between SNPs and STRs, results were compared for 40 individuals analyzed by both markers (*n* = 32 captured and *N* = 8 captive).

### 2.5 Assessment of reliability to estimate relatedness and inbreeding

The selection of the most reliable estimate of relatedness is essential for the genetic monitoring of the breeding program. Relatedness indices were calculated using seven different estimators: five moment‐based estimators (Li, Weeks, & Chakravarti, 1993; Lynch & Ritland, 1999; Queller & Goodnight, 1989; Ritland, 1996) and two likelihood‐based estimators (Anderson & Weir, 2007; Wang, 2007). All above estimators were implemented in the COANCESTRY version 1.0.1.5 software (Wang, 2011). To determine bias and precision of the different estimators, we used COANCESTRY to generate 100 pairs of genotypes for different relationship categories, that is, unrelated (UR), first cousins (FC), half‐siblings (HS), full siblings (FS), and parent–offspring (PO), based on observed allele frequencies at each locus estimated in the broodstock population, which is representative of the genetic diversity of the species (Roques et al., 2018). We choose that option in COANCESTRY that takes into account some level of inbreeding. Relatedness coefficients were estimated for these groups of simulated individuals (PO SIM, FS SIM, HS SIM, and FC SIM) as well for two groups of individuals of known kinship (i.e., empirical and EMP; 36 individuals with full siblings, FS EMP and 16 individuals with parent–offspring, PO EMP). Based on the simulated dataset, COANCESTRY also calculates a matrix of correlation coefficients among the seven different relatedness estimators and the true simulated values. The best estimator is the one that has the highest correlation with expected values, that is, 0.5 for PO and FS, 0.25 for HS, 0.125 for FC, and 0 for UNR (Wang, 2011). Comparisons of relatedness between STRs and SNPs and among relatedness categories were carried out using nonparametric tests (Mann–Whitney or Wilcoxon tests). We also considered the most accurate estimator, the one with smallest interquartile ranges for each category of kinship and with the weakest overlay between relationship categories.

Because inbreeding will be considered for the breeder's selection in captivity, we also evaluated reliability and precision of SNPs and STRs in estimating inbreeding. First, we calculated *F* values in wild‐born breeders (BREEDERS, *N* = 36) and F1 juveniles (F1‐JUV; *N* = 33 juveniles; software KINGROUP; F TrioML and F Dyad ML, for SNPs and STR, respectively). Because the F1 generations produced in captivity are issued from only a subset of breeders, some of them being related, we expect inbreeding to be higher in F1 than in wild breeders because of selection and genetic drift. We used Wilcoxon rank sum tests to test for significance. Recent works have also proposed identity disequilibrium (ID) as a measure that may capture variance in the level of inbreeding within a population (Stoffel et al., 2016). The INBREEDR package (Stoffel et al., 2016) allows the exploration of several parameters to quantify ID (as measured by the *g*2 statistic; David, Pujol, Viard, Castella, & Goudet, 2007). An especially interesting purpose of this package is to test the effects of the number of loci on the precision and magnitude of inbreeding, *g*2, by simulations. We specified the number of simulated individuals to n_ind = 50, the subsets of loci to be drawn (i.e., 1–17 and 1–79 for STRs and SNPs, respectively), the heterozygosity of noninbred individuals (i.e., the expected heterozygosity He in the base population, BREEDERS; of 0.5 and 0.6 for STRs and SNPs, respectively), and the distribution of *F* among the simulated individuals to measure *g*2. The *F* values of the simulated individuals are sampled randomly from a beta distribution with mean (meanF) and variance (varF) specified by the user (i.e., mean *f* = 0.06 and varF = 0.008 for both SNPs and STR). Also, to infer how well genetic marker heterozygosity reflects the inbreeding level *F* and whether this correlation could be improved by using an increasing number of markers, we also use and compare the “simulate_r2_hf() function” among both markers (Slate et al., 2004; Szulkin, Bierne, & David, 2010). We further calculated and compared *g*2 values between SNPs and STRs in wild‐born breeders (BREEDERS) and F1 juveniles (F1‐JUV). A value of *g*2 significantly greater than 0 is interpreted as evidence that those markers contain information about variation in inbreeding.

Finally, we were interested in comparing individual heterozygosity and inbreeding estimates between STR and genome‐wide SNP markers. We explored associations between estimates of inbreeding and heterozygosity based on 76 individuals. Pearson's correlations (“cor.test” in *R*) of the individual inbreeding coefficient *F* and the individual heterozygosity by loci (HL; proportion of heterozygous positions or loci) available in GenAlex (Peakall & Smouse, 2012) were calculated between markers.

Comparison of relatedness and inbreeding using nonparametric tests and Pearson's correlations of individual heterozygosity and inbreeding was carried out using R software (R Core Team, 2013).

## 3 RESULTS

### 3.1 Sequencing results

In total, 92,063,509 reads (from 1,534,526 to 2,853,548 reads per individual) with a median read length of 206 bp were generated through the sequencing of the 40 individuals. After the STACKS filtering steps, a large number of SNPs (*N* = 19,444) were retained for the species (see Appendix S3). The percentage of loci shared across ≥75% individuals was not very high (24.5%; 4,775 out of 19,444 loci) probably due to the low sequence coverage, but reach 42% (8,091 out of 19,444 loci) when the proportion of individuals was set to a lower value (>50% individuals; *N* = 20). When comparing the two technical replicates, we found that 29% of loci was only present in one or the other replicate (mean locus error rate), while mean allele error rate (number of allele mismatches over the total number of loci compared) was of 9.6% (*N* = 454 mismatches/4,742 SNPs).

### 3.2 Single nucleotide polymorphism genotyping assay

From the initial set of 154 SNPs used for the SNP genotyping assay, we discarded monomorphic SNPs (10%), SNPs whose profiles showed two groups of heterozygotes (putative paralogous loci or ancient tetraploidy; 8.8%) and SNPs for which amplification intensity was low in most samples (2.6%). Finally, three additional SNPs were further removed for inconsistency in Mendelian inheritance, checked in family groups. The final set after filtering includes 79 SNPs**.** There was no mismatch between the two replicates that we included in the genotyping analysis (100% concordance). Minor allelic frequency (MAF) varied from 0.214 to 0.489 depending on SNPs (mean = 0.389 ± 0.070; Appendix S4).

### 3.3 Comparison of SNPs and STRs for individual identification and parentage assignment

**Figure 1:**
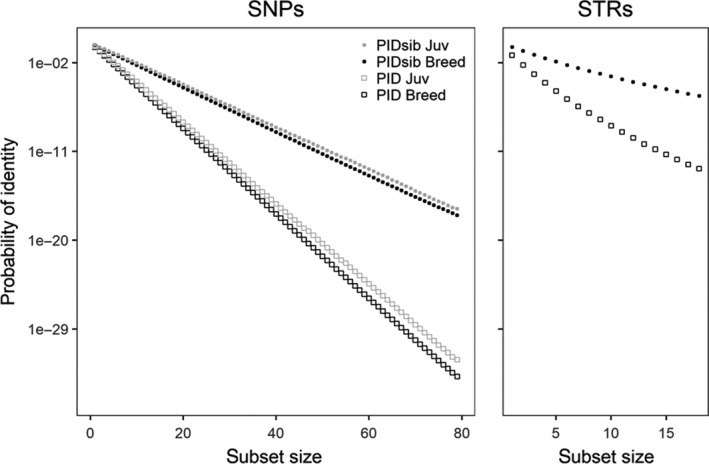
Probability of identity (PID) considering all individuals are unrelated or full sibs (sib), calculated based on the allelic frequencies of the broodstock (BREEDERS; PID Breed) and the future breeders (PID Juv; only for SNPs) for an increasing number of SNPs and STRs (subset size). PID calculations were done using the GenAlEx program

The identification power of the panel of 79 SNPs was estimated based on the PID as calculated in GenAIEx (Figure 1). This panel has a three times higher resolving power for individual identification (PID = 3.4 × 10^−18^ and PID = 1.5 × 10^−34^ for full siblings and nonrelatives, respectively) than the panel of 18 STRs (PID = 4.2 × 10^−6^ and PID = 1.8 × 10^−13^, for full siblings and nonrelatives, respectively), based on the allelic frequency of the broodstock. The best PID value for STRs is reached with only 28 SNPs. The PIDs obtained considering the allelic frequency of the future breeders (F1 juv) are high and very similar to that based on BREEDERS (Figure 1).

Results of parentage assignment in the captive stock of F1 juveniles indicate a high level of assignment for individuals of known parent pairs. All but four (i.e., 97%, 4/128) have been assigned to the parent pairs recorded in the captive stock database of origin with 95% confidence. For three (ID#149, 150, and 209) of these four individuals, the identification has been likely mistaken during sampling and handling in captivity: Individual 149 was genetically assigned to the parents' pairs of individual 150, and individual 209 was assigned to a parent pairs that was also crossed the same year (both assignations with 95% confidence). This may lead us think to family mixing during handling in the tanks at young stages or during tagging procedures. If we accept this “misidentification” hypothesis, genetic assignment then reached 99% success. Among the sturgeons for which only female identification was available (*N* = 66), the assignment of males was highly concordant among the multiple choices for all individuals except for one (ID#297).

Among the 40 samples assigned by both SNPs and STRs, concordant results were observed for 25 individuals (see Appendix S5). Among these, three individuals were assigned to pairs not recorded in the breeding program with both markers, which suggest they could be issued from natural reproduction. Among the 15 nonconcordant results, 13 samples were assigned to nonexisting parent pairs in the breeding plan with STRs (Roques et al., 2016), while SNPs successfully assigned to recorded parents pairs: In 11 of these cases, the candidate father given by STRs was the offspring of the father identified by SNPs. For the remaining two cases, the parent pairs assigned by SNPs and microsatellites belong to different year; thus, we cannot conclude if it is a misidentification or a genetic assignment error.

### 3.4 Comparison of SNPs and STRs for assessment of relatedness, inbreeding, and heterozygosity

Best correlation coefficients (calculated in COANCESTRY) among the seven relatedness estimators and the true simulated values were obtained for SNPs. The correlation coefficients were significantly lower for STR (varied between 0.476 and 0.677) than for SNPs (varied between 0.819 and 0.869; Wilcoxon test *W*; *p*‐value = 0.0006). For both SNPs and STRs, the best correlation was found for maximum‐likelihood (ML) estimators. TrioML and DyadML were considered as the most accurate estimators for subsequent analyses for SNPs and STRs, respectively.

**Figure 2:**
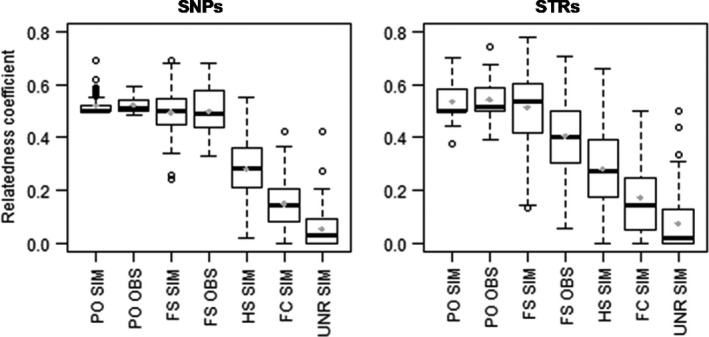
Box plots of relatedness coefficients (DyadML and TrioML, for STRs and SNPs, respectively) for different relationship categories in simulated (SIM; i.e., 100 pairs of simulated genotypes; program COANCESTRY) or empirical (OBS) datasets (see Materials and Methods for details): FC, first cousins; FS, full sibs; HS, half‐sibs; PO, parent–offspring; UNR, unrelated. Gray dots represent the mean for each relationship categories

A gradient of relatedness coefficient relating to kinship was found for both SNPs and STRs (Figure 2). It is worth noting that the relatedness coefficient values for known full sibs (FS OBS; mean *R* = 0.497 ± 0.085 and 0.424 ± 0.149 for SNPs and STRs, respectively) were not significantly different from expectations (i.e., *R* = 0.5; Wilcoxon test, *w* = 16, *p*‐value = 0.92 for SNP; *w* = 9, *p*‐value = 0.45 for SNP). Also, as expected, there were no significant differences (pairwise comparisons using Mann–Whitney tests, *p*‐values >0.05) between the relatedness coefficients of PO and FS categories for SNPs; while using STRs, we observed significant differences between FS OBS and either FS SIM, PO OBS, and PO SIM (*p*‐value <0.01; Figure 2). All other category comparisons for relatedness coefficients were significantly different for both markers, as indicated by *p*‐values ≤ 0.01. The discrimination of kinship categories was better for SNPs than for STRs. The interquartile ranges (IQRs) were significantly lower for SNPs than for STRs (Wilcoxon test, *w* = 0, *p*‐val = 0.02). Furthermore, unlike for STR, IQRs of SNPs did not overlap among the different relationship categories (except for PO and FS which are kinships with similar expected relatedness values).

**Figure 3:**
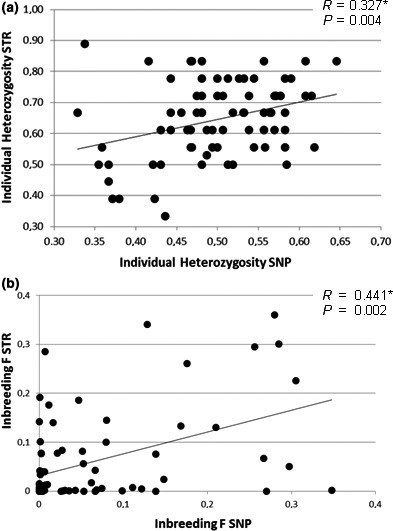
Correlation between SNPs and STRs markers (*N* = 76 individuals) for (a) individual heterozygosity by loci (calculated in GenAlEx) and (b) inbreeding *F* (calculated in COANCESTRY). *Significant after Pearson's correlation test's

We analyzed the relationship between individual heterozygosity *H* (Figure 3a) and *F* estimates of inbreeding (Figure 3b) at SNPs and STRs, and we found slight but positive correlations (*R* = 0.327, *p* = 0.004 and *R* = 0.441, *p* = 0.002 for *H* and *F*, respectively). For both markers, mean inbreeding in F1 juveniles (F1‐JUV; mean *F* = 0.093 SNPs and *F* = 0.088 STRs) was significantly higher than that in BREEDERS (mean *F* = 0.022 for SNPs and STRs; Wilcoxon test; *p*‐value <0.001).

**Figure 4:**
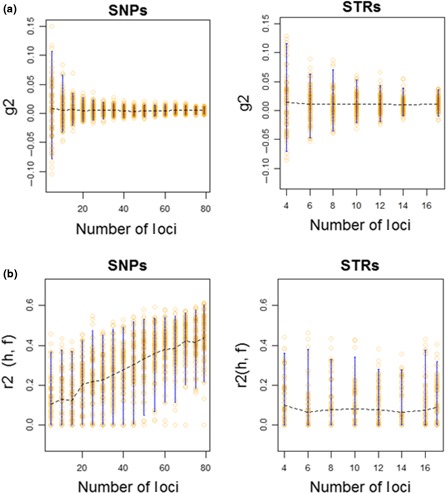
Plots of the distribution of (a) Bootstrapped *g*2 values and (b) expected correlation *r*2 (*h*, *f*) between standardized multilocus heterozygosity (*h*) and inbreeding level (*f*), for the different marker subsets samples (number of loci), including their means (dotted line) and 95% Cis (in blue). Different sets of STRs and SNPs were simulated and drawn from distributions based on inbreeding level *f* and heterozygosity level from *Acipenser sturio* empirical datasets (i.e., BREEDERS)

The analysis from INBREEDR package showed that the variation around *g*2 estimates is higher for STRs than that for SNPs, and decreases when a higher number of markers are used (Figure 4a). The precision on the *g*2 estimates is higher for the set of 79 SNPs than that for STRs, for which variance is high. The expected correlation between inbreeding and marker heterozygosity (Figure 4b) is almost twice for SNPs than that for STRs. The estimate precision is similar for 1–17 STRs, but increases slightly for SNPs when increasing the number of markers used, although we observed that confidence intervals are still quite large for the whole set of 79 SNPs (Figure 4b). The estimates of inbreeding (*F* and *g*2 values) are both concordant in higher inbreeding level in F1 juveniles than wild‐born breeders for both marker types: At SNPs, *g*2 values were slightly positive and significant for F1‐JUV and BREEDERS (0.0095 and 0.0217, respectively; *p*‐value <0.01; evidence of identity disequilibrium) while at STR values (0.0112 and 0.010, respectively) were only statistically significant for F1‐JUV (*p*‐value <0.01).

## 4 DISCUSSION

The use of high‐throughput sequencing techniques in nonmodel organisms has opened the way for obtaining numerous SNP markers that may compensate the limited power of other markers, especially in scenarios of small and/or decreasing populations composed of related individuals. Our study showed that double‐digest RAD sequencing (ddRADseq‐ion; Recknagel, Jacobs, Herzyk, & Elmer, 2015) worked well for the rapid and cost‐effective generation of a significant number of polymorphic and reliable SNPs in a species with very low diversity and with no a priori genomic information, the critically endangered European sturgeon. As well as developing new markers, we also get massive sequence datasets for the whole breeding stock of the species (the origin of all released individuals and future breeders), information that we considered potentially useful in the future.

While the number of novel SNPs that can be discovered might be high, in this study we highlighted the importance of ensuring reliability. The accuracy of genotypes that will be further analyzed for a wide range of applications is crucial but often limited by the occurrence of sequencing errors inherent to high‐throughput sequencing techniques (Mastretta Yanes et al., 2015). The frequency of SNP genotyping errors is not systematically estimated when genotyping; it usually depends on the technology used, but also on the validation steps that precede the final selection of SNPs (Mastretta Yanes et al., 2015). Here, we were particularly careful in applying a very strict filtering protocol (fully described in *Material and Methods*) to ensure the maximum reliability of genotypes although this has consequently reduced the number of loci retained (i.e., 154 out of 19,444; 0.8%). Our proportions of ddRADseq error rates were high between replicates (locus error rate of 29% and allele error rate of 10%) and higher than other similar libraries using Ion Torrent platform (Recknagel et al., 2015) but very similar to that of other NGS platforms such as Illumina (e.g., Mastretta Yanes et al., 2015). These errors are especially relevant in parentage analysis, because they may impede correct assignment and bias results. Congiu et al. (2011), for example, reported that the total lack of correspondence between offspring and parental genotypes determined directly from sequence data was explained by errors in the RAD sequence genotypes of the parents. Our final set of 154 SNPs selected from ddRADseq was validated a posteriori on a higher number of individuals and families using the Agena MassArray system. This approach based on mass spectrophotometry proved to be a highly sensitive, reproducible, and reliable compared to others genotyping technologies (Bradic, Costa, & Chelo, 2011; Gabriel et al., 2009; Miller, Buchner, et al., 2014; Miller, Malenfant, et al., 2014). After validation, a proportion of SNPs which were polymorphic after ddRAD sequencing proved homozygous (10%) when genotyped by the MassArray technology. These results highlight the possible discrepancy between methods in obtaining genotypes, in this case most likely the results of the low ddRADseq coverage. They further underline the importance of including technical replicates and family groups to detect them in the experimental design, as we did here. It is also worth noting that the MassArray system produced fully concordant results between replicates (no mismatch) therefore supporting a high reliability and consistency for this technology that will be used in routine for the genetic monitoring of *A. sturio*.

The main aim of this work was to develop a set of SNPs with higher resolution than STRs and primarily with enough efficiency to resolve situations in which individuals might be closely related. This study is probably one of the most extensive comparisons of STRs and SNPs that estimates reliability and precision on parentage, relatedness, and inbreeding. This work clearly showed that the full panel of 79 SNPs (four times the number of STR loci) was more powerful and reliable than the previous 18 STRs to determine the paternity and identity in the European sturgeon population. Results demonstrated that SNPs gave more accurate identification (i.e., a threefold higher probability of identity, PID) than STRs and that the maximum resolution of STR was achieved using only 28 SNPs. These results are comparable to a study on another critically endangered species, the Iberian lynx, with very low diversity and for which a relatively reduced number of SNPs (*n* = 24) had sufficient power to discriminate even between closely related individuals (Kleinman‐Ruiz et al., 2017). To take into account a more realistic scenario for the future management of the species, in which we will have to resolve paternity of next generations (F2), we tested the sensitivity of SNPs considering the allelic composition of the future captive breeders (i.e., F1 juveniles born between 2007 and 2009). We showed that the power of resolution with this last setting was still very high and almost similar to that based on the original broodstock's allelic frequencies, corroborating the similarity between the patterns of genetic variability of captive breeders and F1 captive stock, as reported earlier (Roques et al., 2018). Also, previous studies have shown that assignment errors are highly dependent on the presence of other categories of kin in the sample (Marshall, Slate, Kruuk, & Pemberton, 1998; Olsen & Vøllestad, 2001; Figure 2). Roques et al. (2016) using STRs observed a low percentage of captured *A. sturio* individuals in the wild that was assigned to parent pairs not recorded in the breeding program. While these results may indicate a wild origin of these individuals (*N* = 20), the fact that the sensitivity of the methodology may be at play could not be excluded. Here, we showed that limited resolution of STRs was probably the key impediment in discriminating among related breeders. Indeed, SNP markers were successful in assigning known parent pairs to 17 of these 20 individuals which reduce the number of fish that might be originated from natural reproduction. For 11 of these individuals, the males identified by STRs are the offspring of the male (Justin) identified using SNPs. In this case, it proved that when candidate parents are highly related (parent–offspring or full sibs), SNPs are better at assigning fathers than STRs.

Another important application of genetic markers in captive breeding programs is breeding strategies based on the minimum kinship criterion, which aims to select the less related parent pairs each year, to reduce inbreeding and retain genetic diversity (Fernández & Caballero, 2001; Fraser, 2008). For the *A. sturio* breeding program, reproduction is assisted so that parent pairs can be selected and recorded from among all possible mature adults and separate rearing of families is carried out until fish reach about 6 months for individual identification with PIT‐tags. This individual identification of all breeders (F1) and future breeders (F2) reduced the probability of crosses among highly related individuals. However, because most of these fish are produced from a small number of families and breeders, some of them being related, progenies are genetically closed. For this reason, it was important to select the most reliable relatedness parameter to avoid an overrepresentation of inbred individuals in the future families. This strategy has been applied in the *A. sturio* breeding program since 2014 based on STR markers (Roques et al., 2018), and this will continue to be the strategy moving forward, based on the novel SNP markers. One straightforward approach to determining the kin relationship in a group of individuals relies on the use of pairwise relatedness estimators, which measure the amount of genetic material shared by descent between individuals. The most appropriate estimate of relatedness may differ for a given set of markers and context (Van de Casteele, Galbusera, & Matthysen, 2001). The kin structure of the population is a very important clue, because any relatedness between individuals is dependent on the level of ancestral relatedness of the given population (Milligan, 2003; Weir, Anderson, & Hepler, 2006) which is generally high in populations of endangered species. If this effect is ignored, relatedness estimates could be underestimated. This is highly relevant for the European sturgeon, since its remnant population is most likely issued from related ancestors (Chassaing, Desse‐Berset, Hanni, Hughes, & Berrebi, 2016; Roques et al., 2018). Our results of the detailed comparison of several relatedness estimates based on empirical and simulated data set of known kin relationship (both highly related and unrelated) indicated that there are significant differences in their reliability and variance. We observed that the two likelihood‐based methods were the most reliable and had the lowest variation in individual pairwise *r* values, while estimates differed for SNPs and STRs (DyadML and TrioML estimators, respectively). Interestingly, the mean relatedness over all pairs of individuals in each kin group corresponds well to the expected pedigree relatedness, but these distributions are overlapping for different kinship categories, especially for the half‐sib category (see Figure 2). Kleinman‐Ruiz et al. (2017) also observed that the discrimination of the half‐sib from unrelated, for example, needed a higher number of SNPs, because it was one of the most demanding comparisons. The distributions observed here, however, were very similar and even less overlapping than those observed in others studies using simulated or empirical datasets (Blouin, 2003; Blouin, Parsons, Lacaille, & Lotz, 1996; Russello & Amato, 2004). This and the other studies underlined the high degree of difficulty of inferring the probability of a relationship given the measure of relatedness between two genotypes, for adjacent categories. Variance in the sharing of alleles by state or inaccurate measures of the population's allele frequencies are among the most frequent reasons invoked for this bias.

In this study, we observed that SNPs performed better than STRs in estimating inbreeding in groups with known expected level of inbreeding (based on relatedness of parents). Inbreeding is another important parameter to measure in captive breeding programs because inbred individuals have lower fitness than the offspring of unrelated parents (Hedrick & García‐Dorado, 2016; Kardos, Luikart, & Allendorf, 2015; Kardos, Taylor, Ellegren, Luikart, & Allendorf, 2016). These results also gave new insights on the sensitivity of markers to detect identity disequilibrium. Single nucleotide polymorphism markers give more reliable estimation of inbreeding *g*2 (less variance) than STRs and sensitivity increases with increasing the number of markers used. Similarly, the expected correlation between inbreeding and marker heterozygosity was also almost twice for SNPs than for STRs and increased when the number of markers was increased (Figure 4b). Based on the above results, the proposed breeding strategy for *A. sturio* (in the short term, mostly including the 2007–2009 captive cohorts) is to build a reliable and comprehensive studbook, which will optimize the retention of diversity and limit inbreeding in the captive and sustained population. This will be done by suggesting potential pairings among the available mature breeders or eventually selecting cryopreserved gametes. Optimum pairings will be based on the mean kinship (i.e., relatedness coefficient TrioML) and by calculating inbreeding coefficients to select the most heterozygous individuals to maximize diversity for releases into the wild or for breeding individuals.

Because of the absence of a reference genome sequence for the European sturgeon, the locations of putative SNPs on chromosomes or linkage between markers could not determine and further analysis would be necessary. The significant positive correlations found between STRs and genome‐wide SNPs in this study for both individual heterozygosity and inbreeding (Pearson correlation tests; *p* values = 0.004 and 0.0021, respectively) may, however, suggest that variation at both markers may reflect genome‐wide genetic diversity. The analysis of large number of SNPs is supposed to provide a greater power and precision to quantify genomic levels of diversity and inbreeding (Kardos et al., 2016). This is because the measurement of variability of SNPs scattered across a significant fraction of functionally important genes should make possible the reliable prediction of overall genetic variation (Vali et al., 2008). Because there have been only a few cases of positive and weak correlations between expected microsatellite heterozygosity and SNP diversity (Payseur & Cutter, 2006; Ryynanen, Tonteri, Vasemagi, & Primmer, 2007; Vali et al., 2008), our results are interesting and diverge with theory that suggests that an association of heterozygosity estimates between STRs and SNPs is not expected a priori (reviewed in Ljungqvist, Kesson, and Hansson (2010)). Indeed, Vali et al. (2008) highlight that STRs have usually provided a poor prediction of the genome‐wide nucleotide diversity of wild populations at the individual level. Ljungqvist et al. (2010) have proposed that a strong positive correlation may emerge when the studied populations are characterized by substantial identity disequilibrium, as shown in a few studies for several species including for salmon (Ryynanen et al., 2007), for the Scandinavian wolf population (Vali et al., 2008), or in wild sheep (Miller, Buchner, et al., 2014; Miller, Malenfant, et al., 2014). While our results indicated only slight correlations, the identity disequilibrium (i.e., positive *g*2) found for the European sturgeon may give an empirical and interesting support to the above theory.

## 5 CONCLUSIONS

The increasing amount of genetic marker information that can be generated by new sequencing techniques should undoubtedly provide better genetic tools and better description of genome‐wide diversity, useful for the conservation of endangered species. The right selection of markers has been discussed in recent papers that have stressed the importance of the type, number, reliability, and genome representability, for an optimum choice, although detailed empirical assessments of such parameters have been scarce (Kleinman‐Ruiz et al., 2017). Because the genetic variability of *A. sturio* produced in captivity (and eventually released for restocking) is low and is expected to further decrease in the future (i.e., due to selection and genetic drift), a suitable genetic tool with high resolution was required for assessing relatedness, inbreeding, and to assess parentage. We present here a highly efficient and reliable SNP panel that could be genotyped easily with reduced cost and typing efforts, thus providing a standardized panel for the exchange of genotype data between laboratories. Regarding the conservation of small or captive populations, there is a great concern for the loss of genetic diversity through genetic drift and inbreeding. In the context of *A. sturio* conservation program, this assay will be useful for the genetic management of the broodstock and further restocking in France, in Germany or for the future reintroductions in other systems. This approach may also prove suitable for other case studies in which highly discriminatory genetic markers are needed (i.e., endangered populations composed of related individuals or populations issued from a small number of founders) and in which the transition to SNP markers is planned.

## CONFLICT OF INTEREST

None declared.

## AUTHOR CONTRIBUTION

S.R. designed the study, performed SNP development and analysis, analyzed and interpreted data for parentage, relatedness and inbreeding assessment, and wrote the manuscript with help from all coauthors. M.L.A supervised the study, coordinated sampling collection, and got funding. E.C. and C.B. designed and performed SNP discovery experiments, analyzed, and interpreted data. M.P. performed all statistical analyses. All authors revised the manuscript critically and approved the version to be submitted. Final approval of the version to be published.

## Data Availability

Genotype database available from the Dryad Digital Repository: https://doi.org/10.5061/dryad.t9v70p3
