# Supplementary material for: From microsatellites to single nucleotide polymorphisms for the genetic monitoring of a critically endangered sturgeon
Source: Ecol Evol. 2019 Jun 11;9(12):7017–29. doi: 10.1002/ece3.5268 (PMC6662312; doi:10.1002/ece3.5268)
Supplement: Supplementary file 2 [file ECE3-9-7017-s002.docx]

**Appendix S2:** SNP filtering workflow from ddRAD sequencing reads on n=40 Acipenser sturio to the design of multiplex SNP for MassArray validation.

**92 063 509 reads on 40 samples (ddRADseq)**

**STACKS (m=6 ; M=2, n=3)**

65 350 SNPs

**1 SNP per tag**

28 740 SNPs

**SNP position < 20 bp and > 179 bp** **discarded**

19 444 SNPs

**SNP on N ≥ 20 samples**

8 091 SNPs

**SNP with MAF ≥ 0.175**

3 011 SNPs

**SNP with same genotype between replicates**

2 397 SNPs

**SNP showing Mendelian inconsistencies discarded**

2 125 SNPs

**SNPs having 34-64% of heterozygotes**

262 SNPs

**SNP present on redundant RAD- tags discarded**

186 SNPs

**MassArrayAssay Designer**

154 SNPs
